# A Psychological Analysis of Stuttering

**Published:** 1916-02

**Authors:** 


**A Psychological Analysis of Stuttering.** Walter B. Swift, A. B., S. B., M. D. Jour. of Abnormal Psychology, Boston, Oct.-Nov., 1915. The author's psychoanalysis reveals stuttering as some vague trouble in the personality. Psychologic Analysis shows stuttering is an absent or weak visualization at the time of speech. This new concept of stuttering as faulty visualization may be called Visual Centre Asthenia. This lack or weakness in visualization accounts for all the numerous phenomena of stuttering in severe, medium or mild cases. A new treatment is indicated.

